# Bullous Wells’ syndrome successfully treated with omalizumab

**DOI:** 10.1111/ddg.15964

**Published:** 2025-12-12

**Authors:** Giulia Ciccarese, William Andrew Rosato, Francesco Drago, Alexandre Raphael Meduri, Francesca Ambrogio, Aurora De Marco, Gerardo Cazzato, Caterina Foti

**Affiliations:** ^1^ Dermatology Unit Department of Medical and Surgical Sciences University of Foggia Foggia Italy; ^2^ Section of Dermatology Department of Precision and Regenerative Medicine and Jonian Area (DiMePRe‐J) University “Aldo Moro” of Bari Bari Italy; ^3^ Casa di Cura Villa Montallegro Genoa Italy; ^4^ Section of Molecular Pathology Department of Precision and Regenerative Medicine and Ionian Area (DiMePRe‐J) University “Aldo Moro” of Bari Bari Italy

Dear Editors,

Wells’ syndrome (WS) is a rare cutaneous disease with an unknown etiology. Given its similarity to bacterial cellulitis and the presence of blood and tissue eosinophilia, it is known as eosinophilic cellulitis. The clinical manifestations of WS are heterogeneous and the histological appearance is not specific.^1^, ^2^ Since the disease course is chronic‐remitting, treatment is usually required.^1^, ^2^

Here, we describe a woman with the bullous variant of WS (BWS) who was successfully treated with an anti‐IgE monoclonal antibody.

A 36‐year‐old woman presented with a 3‐year history of an itchy skin eruption consisting of erythematous papules and urticarial plaques on the trunk and extremities (Figure 1a), sometimes annular in shape (Figure 1b). The patient was diagnosed with chronic spontaneous urticaria (CSU) and received systemic treatment with antihistamines and prednisone (initial dose 0.5 mg/kg body weight [BW]/day), which led to clinical improvement. However, the disease relapsed after steroid discontinuation. During the last year, the skin eruptions have been characterized by pruritic vesicles confluent into bullae on the ankles (Figure 1c). The patient denied drug intake or insect bites.

**FIGURE 1:**
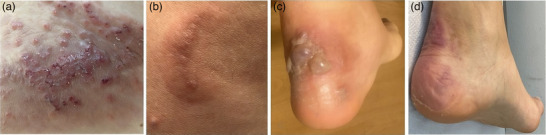
(a) Excoriated papules confluent in a plaque on the patient's back. (b) Erythematous arch‐shaped urticarial plaque on the trunk. (c) Bullae with serous content on the patient's left ankle. (d) Complete resolution of the bullous lesions of the ankle.

Laboratory investigations revealed blood eosinophilia (1.77 × 10^3^/µl; normal range 0–0.53) and elevated total IgE levels (338 IU/ml; normal < 100). Complete blood count, electrolytes, C‐reactive protein, liver and kidney function tests, and complement proteins C3 and C4 were within normal limits. Antinuclear and anti‐extractable nuclear antigen antibodies were negative, as were autoantibodies against the 180‐kD bullous pemphigoid (BP) antigen (BP180), 230‐kD BP antigen, desmoglein (Dsg)‐1, Dsg3, and collagen VII. Parasitological examination of three stool samples and abdominal ultrasound were within normal limits.

Histology from a bullous lesion revealed necrosis of keratinocytes, eosinophilic spongiosis, and a diffuse eosinophilic infiltrate in the dermis associated with some flame figures (Figure 2a–c). Direct immunofluorescence did not show any immunoglobulin or complement deposition.

**FIGURE 2:**
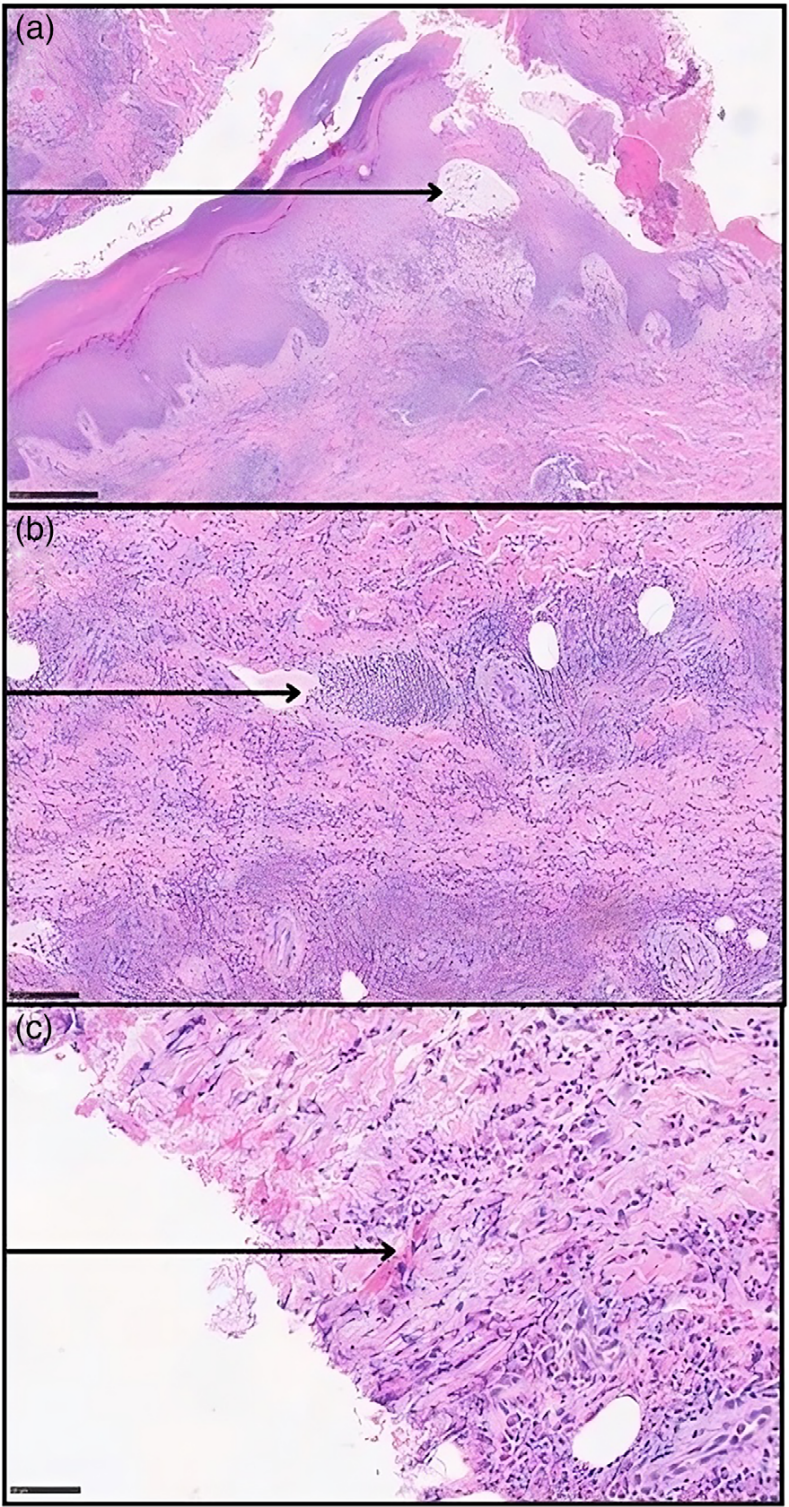
(a) Eosinophilic spongiosis characterized by intraepidermal edema (arrow) with eosinophilic infiltration among keratinocytes (hematoxylin‐eosin stain [HE], original magnification × 4). (b) Diffuse inflammatory infiltrate mainly composed of eosinophils, lymphocytes, and some mononuclear cells in the dermis; the arrow indicates a collection of eosinophils (HE, × 10). (c) Example (arrow) of a “flame figure,” an amorphous eosinophilic structure composed of proteinaceous material and cellular debris, surrounded by degenerated eosinophils and histiocytes (HE, × 20).

Based on the clinical features, laboratory investigations, and histological examination BWS was diagnosed. The patient started therapy with the anti‐IgE monoclonal antibody omalizumab (Xolair^®^, Novartis) 300 mg subcutaneously every fourth week, and she completely recovered in one month (Figure 1d). The total IgE level gradually returned to normal. After one year, treatment was discontinued, but the disease relapsed shortly thereafter. Omalizumab was reinitiated, and efficacy was promptly restored. The patient has been successfully receiving anti‐IgE therapy for 3 years without any reported side effects. Written informed consent for publication was obtained.

Whether WS is a distinct entity or an eosinophilic response to different causative agents is still debated.^1^, ^2^ The difficulty in diagnosing this disease lies in its rarity and its similarity to autoimmune, infectious, or drug‐induced dermatoses. In the case of suspected BWS, several differential diagnoses should be considered: bullous pemphigoid, bullous erysipelas, bullous scabies, bullous drug reaction with eosinophilia and systemic symptoms, Sweet syndrome, and others.^1^, ^2^, ^3^, ^4^ The correlation between clinical, pathological, and laboratory features is crucial for the correct diagnosis.^5^

Although no standardized treatment exists for WS, systemic corticosteroids represent the first‐line therapy, often combined with steroid‐sparing agents such as dapsone or cyclophosphamide in cases of recurrent disease.^1^, ^2^

Our patient is noteworthy as the first reported case of BWS successfully treated with an anti‐IgE antibody, achieving long‐term remission. While a few WS cases responding to omalizumab have been reported in the literature, none involved patients with the bullous variant.^6^, ^7^, ^8^ We hypothesize that our patient initially had CSU and subsequently developed BWS, both conditions known to respond to omalizumab. Although rare, cases of concomitant CSU and classic WS have been documented.^6^ This association should be considered whenever wheals occur concomitantly with long‐lasting, itchy, and painful plaques.

Omalizumab is a well‐established second‐line treatment of CSU, recommended when the therapy with second‐generation antihistamines is ineffective. In the pathophysiology of CSU, skin mast cells are key effector cells: their activation, degranulation, and mediator release are triggered by IgE binding to high‐affinity FcεRI receptors expressed on the surface of mast cells and basophils. Anti‐IgE treatments markedly reduce disease activity by binding free IgE, downregulating FcεRI expression on mast cells and basophils, and thereby preventing their activation.^9^ Several studies have shown that FcεRI is upregulated on skin‐infiltrating eosinophils in atopic dermatitis and bullous pemphigoid,^10^ which may explain the successful use of omalizumab in these eosinophilic conditions. Similarly, it can be speculated that dermal eosinophils in WS upregulate FcεRI, which is subsequently downregulated by omalizumab, leading to clinical remission.^9^

In conclusion, this case report aims to raise awareness of BWS and its available treatment options. Omalizumab appears to be a promising therapeutic strategy.

## CONFLICT OF INTEREST STATEMENT

None.
